# Working it out: Will the improved management of leaky bodies in the workplace create a dialogue between medical sociology and disability studies?

**DOI:** 10.1111/1467-9566.13519

**Published:** 2022-09-05

**Authors:** Jennifer Remnant, Katherine Sang, Katriona Myhill, Thomas Calvard, Sushila Chowdhry, James Richards

**Affiliations:** ^1^ Scottish Centre of Employment Research University of Strathclyde Glasgow UK; ^2^ Edinburgh Business School Heriot‐Watt University Edinburgh UK; ^3^ University of Edinburgh Business School Edinburgh UK; ^4^ School of Health Sciences University of Dundee Dundee UK

**Keywords:** disability, embodiment, employment, leaky bodies, menstruation

## Abstract

This article focuses on the workplace as a significant site of convergence between the disciplines of medical sociology and disability studies. As disability remains on the margins of sociological exploration and theorising relating to health and work, disabled workers remain on the margins of the workforce, subject to disproportionate rates of unemployment, under employment and workplace mistreatment. The article focuses on the experiences of people with ‘leaky bodies’, focussing specifically on employees who experience troubling menstruation and/or have gynaecological health conditions. It brings together data from three studies conducted between 2017 and 2020; interviews with disabled academics (*n* = 75), university staff with gynaecological health conditions (*n* = 23), and key stakeholders in universities (*n* = 36) (including university executives, line managers and human resources staff). These studies had separate, but linked foci, on the inaccessibility of workplaces, managing gynaecological health conditions at work and supporting disabled people at work respectively. Drawing on the Social Relational Model of disability and theories of embodiment, we explore the experiences and management of workers with leaky bodies in UK University workplaces. Data illustrates how workplace practices undermine embodied experiences of workers with ‘leaky’ bodies by maintaining workplaces which ignore their material reality. We highlight that addressing embodied needs alongside acknowledging disabled people as an oppressed political category represents a theoretical meeting point for disability studies and medical sociology.

## INTRODUCTION

This article posits the workplace as an important site of convergence between the disciplines of medical sociology and disability studies. Exclusion from economic participation is a central tenet of contemporary disability studies while medical sociologists have historically theorised sickness as absence from work (Parsons, 1951). There is extensive evidence disabled and long‐term ill people remain on the margins of the labour force, subject to disproportionate rates of unemployment, under employment and mistreatment in the workplace. This is a pressing practical issue, as the incidence of impairment and/or long‐term ill health increases with age, and in the UK, like much of the Global North, there is an ageing workforce whose changing health needs must be accommodated in the workplace.

Though both key disciplines in the theorising of ill health and disability, little research in medical sociology or disability studies has explored in detail the nuanced social and relational experiences of managing symptoms or impairment effects in the workplace. Medical sociology has largely situated employment as one of the many strands of an individual’s biography, liable to disruption resulting from ill health (Bury, 1982) whilst disability studies predominantly focus on the labour market as an important site of political struggle for disabled people (Swain et al., 2013) with little reflection on the embodied experiences of disabled workers. Though both represent key developments from the problematic and essentialist medical model, which rests on unequal power relationships underpinning interactions between ‘patients’ and the medical professions (Blease et al., 2017), in this article we argue theorising in this area can be further developed by exploring the complicated relationship(s) between employment, health and disability.

This article discusses theorisations of disability and long‐term ill health, before moving on to explore theories of embodiment and the dominance of Cartesian dualism in understandings of health and how we might conceptualise the ‘leaky body’. For instance, the more general notion of an ‘unruly body’ arises when ‘there is a misfit between bodily expression and the imposed disciplines of a particular cultural and social environment’ (Hodge, 2014, p. 655). Notions of ‘dirty work’ and gendered/ableist issues within universities are then also outlined to contextualise the management of impairment effects, symptoms and bodily fluids in the workplace.

We present data from three discrete studies, all interview‐based, with various university stakeholders including disabled academics, academics with gynaecological health conditions and those with decision‐making responsibilities with material implications for university employees. The decision‐making participants, recruited as part of study 3, include senior line managers, human resources (HR) representatives, estates staff, research leaders and trade union representatives from the primary union for academics in the UK: University and College Union. Data were analysed independently by overlapping research teams who identified commonalities across the data which are presented in the findings section of this article in three sections: 1) how university policies, practices and processes construct workplace disability; 2) how universities as employers write the material body out of the workplace, and; 3) the continued dominance of problematic notions of what represents the ideal worker in a university context. The article concludes with a discussion of the findings in relation to extant literature and recommendations for how to move forward with a new workplace focussed dialog between medical sociology and disability studies.

## THEORISING DISABILITY AND LONG‐TERM ILL HEALTH

Both disability studies and medical sociology have troubled the dominant model of ill health and disability: the medical model. This is an individualistic model which focuses on curing or fixing the disabled person, to remove what it considers deviant or abnormal (Brisenden, 1986). Many disability activists and scholars have rejected this model, developing the social model of disability, a dualistic model which distinguishes an individual’s impairment or condition from their disablement, which is the oppression they experience as a person with an impairment interacting with an ableist world (Watson, 2002). By situating disability as an environmental, social and cultural issue of exclusion, the social model is a significant political tool for disabled activists and has functioned as an important call‐to‐arms to resist the multi‐faceted oppression experienced by disabled people (Swain & French, 2000).

Despite its political utility, the social model has been critiqued for not allowing greater insight into impairment effects or symptoms of long‐term conditions. Critics have noted how the model makes little room for those who experience pain or debilitating symptoms, including gendered conditions such as endometriosis (Jones, 2016). In challenging the medicalisation and pathologisation of human difference, disability studies and medical sociological literature have been central contributors to building constructionist, social models, and feminist understandings of gender and disability (Egner, 2016). These critiques laid a foundation for the development of critical disability studies, queer and crip theoretical approaches to both disability and sexuality which allow for more complex understandings of lived experiences that lie at the intersection of sexuality and disability (Egner, 2016; Kafer, 2013). Crip theory in particular, has been utilised to great effect to trouble heteronormative assumptions about desirability, attraction and erotica (McRuer, 2011). A criticism of these critical scholarships is their largely abstract and theoretical application, rather than empirical (Egner, 2016). This is relevant to the current article, as the post‐modernist foundations of Crip Theory would have explanatory potential regarding the gendered elements of the data, but can only be clumsily applied to the bounded, procedural world of paid employment.

For this reason, we draw instead on the Social Relational Model of disability (Thomas, 2004), whereby those designated with ‘impairments’ are oppressed by those without but where the individual impacts of impairment effects are still acknowledged. Disablement, within this approach, is the consequence of three combined and interrelated issues; unavoidable ‘impairment effects’ on embodied functioning in the social world; ‘barriers to doing’ which are materially and socially imposed; and ‘barriers to being’ which reflect the negative impact of having a disabled person’s identity questioned and self‐esteem threatened using language (Cologon, 2016).

Social relational approaches are well suited to the nuances of employment relationships (Sang et al., 2016), where disability is assumed to relate to what disabled people cannot do, obliging disabled workers to enact a positive identity around what they are able to do by consistently having to evidence productivity and demonstrate their value (Jammaers et al., 2016). As such, Thomas’ (2004) Social Relational Model of disability also allows for the analysis of the social relational discursive construction of disability for workers, where the disabled worker is constructed in opposition to the often‐unspoken able‐bodied norm (Sang et al., 2016). Importantly, the social relational model of disability is well suited to understanding the complex interactions between organisational management practices and policy, and the resulting disability of workers with hidden impairments (*ibid*).

There is some commonality between social relational models of disability and medical sociological theorisations of ill health due to their shared recognition of impairment effects. However, an important critique of medical sociological work relates to its apolitical and individualised focus, which is informed by the idea of disability caused by illness and impairment and entails suffering and social disadvantage (Thomas, 2004).

## THE LEAKY BODY

It is medical sociological interest in the corporeal body that has led to improved understanding of embodied experiences of ill health, pain and disability, and given rise to the notion of a ‘leaky body’ (Shildrick, 1997). Shildrick (1997) problematised the medical model of the body which is premised on the (Cartesian) dualist notion of a so‐called mind/body separation. The mind according to the Cartesian model assumes superiority as a ‘thinking substance’ associated with reasoning and selfhood. In contrast, the corporeal body is reduced to a mechanistic substance governed by cause and effect. Leder (1990) suggests it is only when our body fails to function as expected does it come into our awareness. This is particularly so for the unbounded body (Lawton, 1998) where leaking or oozing fluids such as blood, urine and faeces represent matter out of place (Douglas, 1966), disrupting the social order. Further troubling the cartesian dualism mentioned above, Price (2015), reflects on the necessity of conceptualising disability as a bodymind experience. She argued that feminist theorising of disability must pay attention to pain. The gendered implications of leaking can be directly associated with pain (Jones, 2016: Price, 2015) and fatigue (Yunus, 2002) because they are caused by numerous conditions which are exclusively or disproportionately experienced by women and are often experienced comorbidly. A central critique of medical sociology in this article, is how there has been little exploration of the (mis)management and (lack of) support available for those managing physical unruliness or leakiness resulting from menstruation and associated gynaecological health conditions in the workplace, despite the centrality of paid work in UK society (Beck et al., 2021).

Sang et al. (2021), introduced the concept of ‘bloodwork’: the additional labour carried out by workers who menstruate in the containment, management and masking of menstrual blood and gynaecological health conditions in the workplace. This work contributes towards the growing body of organisational studies literature recognising the body as a key material aspect of how labour is organised, and how it affects lived experiences of work for specific workers including women experiencing menopause (Jack et al., 2018) and pregnancy (Gatrell, 2013). However, such extant research often focuses more on organisational responses or individual subjectivity of women, rather than the management of material bodily effects of menopause and pregnancy, such as bodily fluids. Though acknowledging the medicalised, physiological experiences of women, notably little research has explored the experiences of disabled workers or workers with long‐term health conditions. For the purpose of this article, informed by extant research and data presented, we take a broad definition of what a ‘leaky body’ can be, inclusive of those who experience an urgent need to use the toilet, are incontinent and/or menstruate. Though these impairment effects do not necessarily equate to disability, those that experience them are disabled by a working environment that does not adequately accommodate their needs, as is discussed in the findings sections. We also incorporate pain and fatigue into our understandings of a leaky body, acknowledging that they are often experienced in tandem.

### Dirty/body work

Though few scholars have done so, there is a link to be made between the management of leaky bodies in the workplace, the under and unemployment of disabled people and conceptualisations of work. The concept of body work describes work carried out either on one’s own body or the body of others. Body work may involve ‘appearance work’ which describes work performed with the purpose of enhancing corporate cultural acceptability. The labour required to produce an ‘acceptable body’ is socially and culturally embedded (Black & Sharma, 2001). Body work involving work carried out on the bodies of others may involve varying levels of intimacy, for example, beauty/nursing/sex work. A strand of which includes ‘dirty work’ involving tasks which evoke a visceral response such as disgust or repulsion (Hughes, 1951). Dirty work has an ‘occupational taint’, resulting from the nature of the physical substances and materials workers are exposed to or encounter. Dirty work can also include ‘body work’, which focuses on the efforts required by workers to ensure their bodies meet the expectations of employers (Wolkowitz, 2002, p. 497).

The work of managing one’s own body has important temporal, spatial and gendered elements. The body needs to be attended to differently in different contexts, stages of life and environments, and the management of leakiness is likely to look different in the workplace than the home (White, 2021). Bodily functions cannot be postponed or managed to a place or time more convenient to the worker and facilities in the workplace are unlikely to be as accessible as they are domestically. Leakiness can also be gendered with considerable social shame still surrounding women’s reproductive systems and menstrual blood (Gatrell, 2019). Disabled people, particularly those with leaky bodies, are problematically situated as the bodies on which other people engage in body/dirty work, rather than the managers of their own bodies, or paid workers themselves (Ostaszkiewicz et al., 2016). This article moves away from a workforce engaged in the management of other people’s bodies, and instead focuses on a white‐collar workforce, academics, usually assumed to be far removed from the concept of dirty work, to explore the personal, embodied experiences of managing only one’s own leaky body at work.

## WOMEN AND DISABLED ACADEMICS

Women and disabled people are both underrepresented in senior positions in universities and face a range of obstacles in their day‐to‐day working lives (See Fernando & Prasad, 2018; Brown & Leigh, 2018). Contemporary academia can be characterised by intensive work, long working hours, and rigid notions of the ‘ideal’ worker, who is able to dedicate themselves entirely to work without social, personal, or embodied distraction or deviance (Sang et al., 2015). Academic labour distribution is also gendered: there is evidence women in UK academic workplaces are more likely to be allocated pastoral care, teaching responsibilities, administrative tasks and consequently must work extra hours (Todd et al., 2008) which may contribute to negative health outcomes including stress and burnout (Watts & Robertson, 2011). Some academic women will also have to engage in ‘bloodwork’ (Sang et al., 2021). Despite increasing research documenting gendered disparities in academic workplaces, little has extended to the experience of disabled women academics or those with long‐term health conditions.

Research conducted within other white‐collar working contexts illustrates the link between gender and disability in the workplace, where disability can undermine the patriarchal dividend experienced by men whilst creating further disadvantage to disabled women (Sang et al., 2016). Extant research suggests disabled academics must engage in additional labour to accommodate their personal care requirements (Williams & Mavin, 2015). This includes negotiating workplace adjustments and managing their impairment effects while working to match the performance of their non‐disabled colleagues (Waterfield et al., 2018) who are prioritised within university processes (Taylor & Shallish, 2019).

University employers offer an interesting setting to explore the experiences and management of leaky and deviant bodies in the workforce. Practically, universities promote clear institutional values and missions that invariably have equality, diversity and inclusion at their centre and can offer a point of comparison for the actual lived experience of their workers. Further, university employers can be understood as key institutions in the maintenance of a cartesian style dualism, focussed as they are on mind‐work, not bodywork. This article aims to address a significant gap in the research, namely, how leaky bodies are managed in the workplace by workers themselves and those with material decision‐making powers over those workers. In advancing our understandings of how disability and long‐term ill health are both constructed and managed in the workplace, we argue it is possible to develop theorising inclusive of the politicisation of disability studies and embodied understandings of medical sociology. The following section outlines the methodology and analysis drawn on for this article.

## METHODS

This article brings together the interview data from three connected studies which ran parallel between 2017 and 2020. Study details are summarised in Table 1. Further information on the recruitment, data collection and analysis of each study individually is available in the appendix.

**TABLE 1:** Summarised information on empirical data used in this article

Study	Method	Sample size
Study 1: Understanding disabled academics’ experiences of navigating the academy	Interviews with disabled academics 2017 across the UK. Themes covered: Career history, impairment history, experiences of work and how they could be better supported by employers and trade unions	Interviews *n* = 22 Asynchronous interviews *n* = 53
Study 2. The lived experiences of managing an academic career and a gynaecological health conditions	Interviews with academics at different career stages (2019). Themes explored included” experiences of gynae health including diagnosis, the perceived effects at work and on careers, help sought	Interviews *n* = 23
Study 2: Understanding the needs and perspectives of key stakeholders who develop and implement policy in relation to disability	Interviews with key stakeholders in 3 research intensive Scottish Universities (2019). Themes covered: How participants defined disability, their experiences of managing or supporting a disabled staff member or staff with a long‐term health condition, and what they thought might be the barriers to inclusion for disabled colleagues	Interviews *n* = 35

### Participant characteristics

Study one was conducted with disabled academics (*n* = 75). Each participant was provided with a consent form with study details and interview questions. To accommodate the complex needs of some participants, interviews took one of two forms: (1) twenty‐two synchronous telephone or Skype interviews; or (2) fifty‐three electronic interviews via email or online, shared documents for example, Google Docs. The latter data collection method meant participants could take part while allowing for impairment effects. Seventy‐five people participated in the research and constituted the final sample. The 22 synchronous interview participants from study one are summarised in Table 2.

**TABLE 2:** Characteristics of study one participants

Participant pseudonym	Gender	Age	Impairment	Discipline
Ann	Female	40s	Dyslexia	Social Science
Ainsley	Gender queer	20s	Mobility, gynae, mental health and Asperger’s	STEM
Alpana	Female	20s	Cerebral palsy	Social Science
Alison	Female	20s	Visual impairment	Humanities
Arrabelle	Female	40s	CFS/ME	Social Science
Chloe	Female	40s	Stammer	Social Science
Catherine	Female	50s	Vestibular disorder	Social Science
David	Male	30s	Dyslexia	Humanities
Drew	Male	Not given	TBI	Social Science
Frances	Female	60s	Dyslexia	Humanities
George	Male	60s	Depression	STEM
Harriet	Female	30s	CFS/ME	STEM
Kalie	Female	30s	Mobility impairment	STEM
Louise	Female	50s	Cerebral palsy and mental health	Social Science
Lindsay	Female	20s	Dyslexia	STEM
Leanne	Female	30s	Dyslexia and depression	STEM
Paul	Male	20s	ADD and dyslexia	STEM
Rosie	Female	30s	Neurological and mobility impairment	STEM
Richard	Male	20s	Dyspraxia	STEM
Susan	Female	30s	Neurological	STEM
Scott	Male	60s	TBI	Humanities
Tina	Female	50s	Asperger’s	Social Science

Abbreviations: ADD, Attention Deficit Disorder; CFS/ME, Chronic Fatigue Syndrome; STEM, Science Technology Engineering Mathematics; TBI, traumatic brain injury.

Study two was conducted with university staff with gynaecological health conditions (*n* = 23) and participant characteristics are summarised in Table 3.

**TABLE 3:** Summary of participant characteristics for Study two

Participant	Age	Nationality	Gender	Length of interview	Identifies as disabled	Condition
1	33	White ‐ EU	Female	35 min	Not in UK	Endometriosis
2	52	White ‐ British	Female	33 min	No	Polycystic ovaries
3	54	White ‐ British	Female	46 min	No	Fibroids
4	30	White ‐ British	Female	37 min	No	Vulvodynia
5	45	White ‐ British	Female	62 min	No	Endometriosis
6	N/A	White ‐ British	Female	39 min	No	Endometriosis and polycystic ovaries
7	46	White	Female	26 min	No	Endometriosis
8	39	UK ‐ British	Female	47 min	No	Endometriosis and Fibroids
9	34	White ‐ British	Female	32 min	No	Endometriosis
10	49	White Caucasian	Female	46 min	No	Perimenopause
11	49	Scottish	Female	34 min	Yes	Hormonal issues
12	30	White British	Female	44 min	No	Endometriosis
13	38	White	Female	32 min	No	Endometriosis
14	42	White	Female	40 min	Yes but does not disclose	Pre‐menstrual depression
15	47	Scottish	Female	72 min	Yes	Menopause
16	32	White‐Anglo European	Woman	39 min	No	Heavy menstrual flow
17	40	White – Non‐UK	Female	48 min	No	Endometriosis
18	47	White ‐ British	Female	31 min	No	Fibroids
19	53	White ‐ British	Female	21 min	No	Menopause
20	31	White ‐ British	Female	35 min	No	Heavy menstrual flow
21	42	White ‐ Irish	Female	66 min	Yes	Polycystic ovaries and PMSD
22	53	White ‐ British	Female	24 min	No	Heavy periods
23	26	White ‐ British	Gender Queer	40 min	No	Endometriosis

In Study three other university workplace stakeholders (*n* = 36) (including university executives, line managers, human resources staff and trade union representatives) were interviewed. Their characteristics are summarised in Table 4.

**TABLE 4:** Summary of interview participant roles and organization for study three

Org A	
Wendy	Health and Safety
Anders	Research/Admin
Dala	Research leader with EDI responsibilities
Enya	Research Leader with EDI responsibilities
Olive	Research Leader
Oliver	Research Leader
Quentin	Head of Department
Robert	Head of Department
Deidre	Head of Department
John	Head of Department
Oliver	Research Leader
Anna	Disability Advisor
James	Human Resources
Carina	Trade Union
Queenie	Trade Union
Org B	
Rachel	Research/Admin
Rebecca	Disability Advisor
Clara	Research/Admin
Celia	Disability Advisor
Don	Disability Advisor
Adam	Estates
Catherine	Estates
Pamela	Head of Department
Rahim	Head of Department
Damien	Head of Department
Justin	Head of Department
Victoria	Human Resources
Vanessa	Human Resources
Isaac	Human Resources
Justine	Human Resources
Ursula	University Management
Uther	University Management
Udo	Trade Union
Ian	Trade Union
Arthur	Trade Union
Org C	
Jennifer	Trade Union

Participants in studies one and three were provided with pseudonyms and participants in study two were recorded numerically. They are presented as such in this article. It is important to note that while there are three sets of data used for this study, the three studies were closely interconnected although did not use the same participants. Study 1 revealed a range of issues associated with leaky bodies at work, particularly with endometriosis (heavy bleeding and painful menstruation). This finding then led to study 2, as it was apparent there was a need to explore gynaecological health in more detail, given its relative absence in the literature. While studies 1 and 2 highlighted the lived experience of academics living with disability or long‐term conditions, it was then necessary to understand how these lived experiences could be understood within the context of the organisational management practices within Higher Education in the UK. This is of particular importance for the social relational model of disability, as previous research has shown the impact of management practices in constructing both disability and gendered inequalities for neurodivergent workers (Sang et al., 2016). Consequently, we then moved to interviewing key stakeholders in order to understand management attitudes (a key aspect of the social relational model of disability), policies and practices in the management of workers with leaky bodies.

Participants were recruited for each study based on relevant selection criteria, with each group sharing several key characteristics in relation to their current or recent employment within the higher education sector. Participants from studies one and two identified either as disabled or as having a gynaecological health condition thus providing a key insight into the lived experience of work for disabled people and those managing long‐term health conditions. Participants from study three were representative of other University workplace stakeholders who oversee and enforce formal workplace policies and processes relevant to the management of health at work. The varied perspectives of the participant groups allowed the research team to explore the social and relational experiences of disabled academics, especially about workplace decision making and subsequent material implications. A limitation of all three studies is that researchers did not track demographic data relating to the race or cultural background of participants. We recommend further research that does include this information in acknowledgement of racialised practices and histories that relate to how leaky bodies are conceptualised (Colloredo‐Mansfeld, 1998).

Recognising the ‘not about us, without us’ maxim of disability studies (Charlton, 1998), the research team included, and was led by disabled people, people managing long‐term health conditions including gynaecological health conditions and people with caring responsibilities. As the studies were disability led we were able to secure access to participants who were otherwise reluctant to discuss their health with a non‐disabled person. For study 1 there were a number of interviewees whose participation was contingent on the interviewer’s disability status. As such the research team are not disinterested observers of the phenomenon of disability, rather our positionality enabled greater access to participants and the building of rapport, particularly with disabled participants. All three studies secured ethical approval from Heriot‐Watt University prior to data collection. All participants were assured of their anonymity, and resulting transcripts were accordingly anonymised to ensure individuals and their employers were not identifiable.

### Recruitment

Study one recruited via convenience sampling using social media (Twitter) and a circulation of calls for participants by university heads of schools and disability services in the UK. Study two participants were recruited via a follow up email following completion of a survey related to women academic’s experiences of managing menstruation and gynaecological health at work, detailed in a further article (Sang et al., 2021). Participants to study three were purposively sampled and recruited via direct email using eligibility criteria based on job role and decision‐making responsibilities.

### Interviews

The second author was Principal Investigator or Project Director on all three projects and conducted fieldwork for study one. The third author conducted the fieldwork for study two, and the first author conducted the fieldwork for study three. Studies included synchronous and asynchronous interviews to reflect participants’ preferences and needs. Synchronous interviews were conducted online using video calls, by telephone or in person (all data was collected prior to the 2020 outbreak of COVID‐19) and lasted between 13 and 90 min. The use of interview was in keeping with medical sociological methodological traditions to access full and detailed accounts of participant experiences (Charmaz & Belgrave, 2012). All three studies used a semi‐structured interview approach to ensure participants were able to share their personal lived experiences. In studies one and two participants were asked about their career trajectories, their aspirations, barriers to inclusion and experiences of academic work. In study three, participants were asked about their personal understandings and experiences of disability and illness, and then asked about how they had, or would support disabled employees and/or employees with long‐term health conditions. They were prompted to comment on the policies they drew on, the employment outcomes of their employees and their views on the low disclosure rates of long‐term ill health and disability within academia. The interview questions were drawn from the literature. Transcription was completed within the research team and/or a secure transcription service. Data were initially coded as per the research questions of the discrete projects (Appendix).

### Analysis

This article draws on a thematic analysis of the combined data which allowed for the identification and reporting of recurring themes without being constrained by an existing template as other qualitative approaches might dictate (Braun & Clarke, 2006). We adopted the approach recommended by Kirwan et al. (2017) in managing data analysis across a multi author team with multiple data sets. The first author engaged in a close reading of all the data sets. While data set 1 was analysed initially by author two and author one, data set 2 by author two and author three and data set 3 by author one, team discussions over a series of time points revealed the research team had reached similar conclusions across data sets.

As such, the analysis of this article resulted from overlapping research teams discussing data analysis and identifying matching themes across the datasets. The authorship team has combined expertise and experience in both disability studies and medical sociology as well as organisation studies and management and was able to identify a lack of theorising to account for the experiences relayed by project participants. Where there was apparent mistreatment in the workplace, data appeared more complicated than the political stance taken by social model scholars, but the workplace as a relational site of political struggle was bypassed by various medical sociological theories. Dirty work and leaky body literature were explored after data collection, and as such all elements of this article are empirically informed. The thematic analytical process then, can be considered abductive in approach, in that the development of the themes was informed by a ‘practical compromise of induction and deduction’, capturing the process by which the subsequent theorising occurred (Shepherd & Suddaby, 2017, p. 79).

The primary objective throughout analysis was to accurately represent the subjective viewpoints of the interviewees from all three studies and identify commonalities of experience. Once data collection was complete, the chosen method of analysis for this research was thematic coding. In the first instance the research team familiarised themselves with the data and generated initial codes and grouped themes. To develop this article the authorship team revisited the data to construct further thematic networks across the data sets which were integrated into shared themes. The analytical categories derived from the combined data centre on: (i) how intersecting policies, practices and environments construct disability in UK academic workplaces and limit the availability of management and support; (ii) how the body was written out of university policies resulting in the labour of masking and containment, and (iii) how sexist and ableist notions of what an ideal academic is inhibited the discussion and recognition of the material experiences of disabled academics and academics with leaky bodies. The position of the lead author as an ‘insider’ in relation to occupation and health status and thus the possession of apriori knowledge as to the relationship between health and employment assisted in the analytical process and in particular, the identification of common themes across the different data sets. In addition, the research team across all three projects worked in close collaboration verifying the coding of the lead author, which facilitated discussion regarding the theorisation of leaky workers.

## FINDINGS

Data from all three studies illustrated how workplace practices of UK University employers undermined the embodied experiences of workers with ‘leaky’ bodies by maintaining intersecting sexist and ableist barriers, limiting meaningful inclusion or career progression for disabled participants. Participants experienced the shame and lack of visibility identified in broader research relating to disability and the completion of some dirty/body work (Sang et al., 2021) alongside embodied experiences of leakiness and pain. Below, we present three of the ways in which those with unruly, leaky bodies were limited in the workplace. Firstly, via actively disabling practices, policies and professional norms. Secondly, through the cartesian dualism present in university policies and assumptions writing out the corporeal reality of living and (mal)functioning bodies. Lastly, we present how academic workers are set in competition with the impossible standard of the ‘ideal academic’ which particularly disadvantages women and/or disabled workers engaged in dirty, body work.

***Disabling Practices:*** *how intersecting policies and practices construct disability in UK academic workplaces and limit the availability of management and* *support*.

Data from all three studies highlight the presence of numerous disabling practices in UK university workplaces. Ableism was embedded in all elements of university management and governance structures. For example, data from decision‐making participants highlighted how there were no procedural policies to draw on to adequately support disabled colleagues. Though all the universities represented in the data set had Equality, Diversity, and Inclusion (EDI) statements or policies, these did not offer step by step guides, or offer institutional pathways for accessing support. Instead, as acknowledged by Dala, a senior manager in a university Science department, the policies managers had available to them situated disability or ill health as a problem of absence or performance, not one of diversity or support for unruly bodies:

I mean, it very much is the ones around sickness, absence, attendance, and then unfortunately there’s capability to work, it is those policies.

(Dala, Senior manager, study 3)

In not acknowledging the corporeal reality of workers throughout policy and subsequently, process, universities ultimately denied the flexibility necessary to accommodate problematic bodies that leak, experience pain or fatigue in the workplace. The below participant reflects on the limited options available to her to manage her leakiness:

I had to call in sick because I was leaking through dressings at such a fast rate that I could not teach in the space we were using, with students surrounding the instructor on all sides, including behind….

(Associate Professor, Full‐Time Open‐ended Contract, Study 2)

Many participants discussed their concerns about scheduling, and the requirement they be available for students. The below participant outlines how the combination of limited resources (problematic for all academics, but more manageable for non‐disabled academics), scheduling and few opportunities to discuss problematic leaking created an inaccessible workplace for her:

And then my academic career was taking off and I was doing long hours lecturing. And that was really difficult because of course as you know if you’ve got a 10 o’ clock lecture on Tuesday morning regardless you need to be there and deliver that lecture. And I remember having quite tough times with heavy periods, not feeling well or particularly supported by my work environment for dealing with that…no flexibility with timetabling or with just being able to say “look, could somebody cover for me?” We’re so under‐resourced in academia, I think all universities are, then that’s very difficult to ask for that kind of support.

(Participant 19, female, 53, study 2)

Whilst the flexible nature of academia is often cited as a key benefit for individuals with long‐term health conditions, this flexibility also undermined the efficacy of absence management frameworks in meeting the health needs of employees. Individualised workloads, and lack of cover meant, for the below participant, managing her leaky and painful body resulted in increased levels of stress:

this is where the flexibility of academia is like a double‐edged sword really, because you stay at home or you might call in sick because you know you’re not working. However that work didn’t get done. So you’re lying there anxious thinking “I’m going to have to do this work at another time. No‐one else is going to do it for me. No‐one’s going to write your paper or yeah, carry out your fieldwork. It’s down to you.” So you never, when you’re off sick, you’re never off sick and very accepting of it. With that comes a lot of stress I think.

(Participant 5, female, age 45, study 2)

Similarly, flexibility was also limited for individuals engaged in teaching activities because of fixed timetabling. The difficulties in foreplaning in relation to fluctuating symptoms and ill health are demonstrated in the following quote,

Lots of that stuff is sort of being taken away. I mean, for example in our university, I was just about leave from sorting out your *[own]* timetable with your boss to the university doing it. So it will be utterly random, which will be rubbish for parents, rubbish for anyone with illnesses, so I know that’s kind of a hard thing to ask for but I would think, yeah, flexible timetabling, flexible working hours would be probably one of the biggest ways you could help people with any kind of pain really.

(Participant 9, age 34, study 2)

Participants were able to express how various working practices, particularly around scheduling, limited their ability to ask for tailored support for their conditions, symptoms and leakiness. They described a policy framework discouraging sickness absence or flexibility related to sickness.

***Removing the body from work:*** *how the deviant/dirty body is written out of university workplace design resulting in the labour of masking and containment*.

An important facility in the support and management of a leaky body is the toilet. It was a central concern of participants with leaky bodies and disabled participants, who outlined the numerous ways in which toilets featured in their working lives. Though toilets were largely available to participants in their workplaces, they identified how they were made inaccessible by organisations that did not recognise leakiness, unruliness and/or disability has a holistic impact on an individual’s working life. A useful example is that of Rebecca, study one, for whom access to a conference was made so onerous, that using a toilet became impossible. She used a wheelchair, but having arrived at the venue, she discovered it was only accessible by stairs. To deliver her own talk, she ‘…*had to walk the last 20 metres’* and it took her *‘…40 min to get in ‐ couldn’t duck out to get to the toilet for example.’* For colleagues experiencing leakiness alongside mobility impairments this would render the conference entirely inaccessible.

Rebecca’s experience reveals the implicit assumptions of those who design the built environment in which academics (and other workers) undertake their work. Rebecca reveals how unruly, or leaky bodies are excluded from a key social practice within academia – networking at a conference. Rebecca’s experience was reflected across data from study one, with several respondents indicating access to toilets (or lack of access) was a significant restriction on their attendance at career enhancing meetings and conferences, particularly for those with irritable bowel syndrome and heavy periods.

Enya, below, provides a further illustrative example of how an organisational lack of recognition can have material implications for unruly, material bodies. She explains, rather than increasing the number of accessible toilet facilities to better accommodate gender diversity within the workforce, her employing institution instead reappropriated existing toilets. This again highlights how the embodied experiences of employees are made invisible even in the implementation of EDI strategies:

One of the biggest issues I have come across is toilets, this is conflated with the need to have non gender specific toilets within buildings and therefore… and that is the way of ticking the box within our buildings because then people are losing a disabled toilet.

(Enya, Research leader with EDI responsibilities, study 3)

This was replicated in other EDI strategies, such as the Athena Swan University charter, which is a framework used across the globe to support gender equality within higher education (HE). Participants highlighted how the charter, and charter related activities, provided no meaningful support for women with leaky bodies associated with their reproductive health. The below participant outlines her frustration at how even in forums dedicated to gender inequality in academic workplaces, the embodied experiences of leakiness were not recognised:

Yet we’re not tackling the big issue of that affects women, that affects all women right across every subject in academia which is that women will have periods, women will have pregnancy and miscarriages and all of those reproductive [issues]. And they’re not discussed and they’re not talked about, and I think they are really quite an issue.

(Participant 10, female, 49 study 2)

***Far from ‘ideal’:*** *how sexist and ableist notions of what an ideal academic is inhibit the discussion and recognition of the material experiences of disabled academics and academics with leaky bodies. Ideal academics do clean work and do not have to look after their own bodies*.

For participants across the studies an unruly body conflicted with being an ideal academic, as the ideal worker does not leak, or conduct the dirty work of hiding or managing leakiness. For some this perception was internalised to the extent they concealed their symptoms to ensure others would not find out or see them as unprofessional. As the participant below details, there was a tension for some of the women in our data as they worried their concerns with the materiality of the leaky body were counter to feminist theorising aimed at decoupling sex and gender:

This is so kind of anti‐feminism, I wouldn’t want to be problematic or it’s something for me to manage and I don’t agree with this at all but this is kind of how I approach it, it’s something for me to manage and not kind of have to enforce that on anyone else. And yeah, so I suppose it’s more about my professional credibility perhaps.

(Participant 16, female, 32, study 2)

We can see from Participant 16, and many in our other studies, the management of the leaky body was a matter for the individual, rather than evidence of systematic oppression of workers whose bodies are unruly or leaky. Our data also revealed the leaky body, in relation to problematic menstruation, was associated with fears of appearing unprofessional and unclean in front of students:

I don’t walk between the students… I’m scared my menstruation blood and scent is too strong.

(Research Assistant, Part Time and Temporary Contract, Study 1)

The wider data from the interviews with academics (studies one and two) revealed very few participants had disclosed their impairment(s) to their employers or to students, for fear it would be associated with poor student evaluations and either loss of a job (particularly for casualised academics) or the stagnation of career progression. Such was the extent of the internalisation of these fears and the normalisation of the disembodied academic, several participants in study one stated they would not pursue a further career in academia as they felt it was not possible to be an academic with a leaky body. This was particularly acute where academic work involved field trips where there was little to no access to toilets.

A leaky body, particularly when managing problematic menstruation, affected women during professional engagement with colleagues, for example, during meetings, with some participants fearing this made them appear unprofessional to colleagues:

Heavy bleeding means that I need to leave meetings for breaks before they are over, that I plan my days around access to toilets, not having a private toilet for adequate washing… needing to leave teaching situations in order to change sanitary wear.

(Librarian, Part‐Time, Open‐Ended Contract, Study 2)

## DISCUSSION AND CONCLUSIONS

This article highlights how managing a leaky body can be interpreted as dirty body work and is not included within institutional norms and practices for employees of university employers. Data presented show university workers with unruly, leaky and/or disabled bodies occupy a complicated position within the academic workforce. Limited by organisations that do not recognise the embodied experiences of workers other than in relation to absence or performance, leakiness is at odds with the ideal academic. A key implication of this study and analysis is that organisations should not default to a dated but still dominant model of ill health and disability; one which situates disability and ill health in opposition to the completion of paid work and maintains a Cartesian model for the academic workplace.

The Social Relational Model of disability (Thomas, 2004) allows for an understanding not only of impairment effects, but also how the disabled worker is constructed in opposition to the non‐disabled worker. Our data has advanced this work to reveal the extent to which disabled academics themselves have internalised individualised approaches to disability which locate the problem with them and their own bodies. This is similar to work by Sang et al. (2016) who highlighted that neurodivergent workers did not consider themselves disabled as they were not wheelchair users. In the current study, this internalisation of disability oppression and exclusion operated deeply enough that some participants were considering either withdrawing from academia or not going for promotion as they felt it was impossible to advance or even be an academic with a leaky body. Importantly, we can see how academics involved in work which removes ready and easy access to toilets felt their exclusion particularly acutely.

We see an inherent contradiction between the ideal academic who can work long hours in competitive and high‐pressure environments (Sang et al., 2015) and academics with leaky, painful bodies. Fotaki (2013) demonstrated women’s bodies are not wanted in academia as illustrated by the systemic exclusion of mothers due to the organisation of work in universities (Huppatz et al., 2019). Our data advances these arguments moving away from the identification of women’s bodies with motherhood in academia, towards understanding how the gendered, painful, leaky and unruly body is contra to the disembodied ideal academic.

Our data also revealed gender equality schemes within scientific organisations such as Athena Swan excluded or gave minimal attention or weight to the materiality of women’s bodies. As Participant 16 (study 2) articulated, recognising the effect menstruation may have on women’s careers may create a tension within traditional feminist models which sought to decouple women from biological essentialism. As Jones (2016) and Shildrick (1997) have argued, there is a need to understand the corporeality of women’s bodies if we are to address persistent gendered inequalities. By drawing on the Social Relational Model of disability, we are able to incorporate the impairment effects (for example, bleeding through clothes) with discursive constructions of disability to understand the perpetuation of disability oppression. In line with previous research (Sang et al., 2016), our data shows how the social relational model of disability (Thomas, 2004) and the social relational model of gender (Connell, 1990) are important to understand together. The experience of menstruation and associated leaky bodies in the workplace draws attention to the gendered nature of workplace experiences and disability in university workplaces.

However, the Social Model of disability, which has become the dominant model of disability in the UK, while useful for relocating disability as a form of oppression, does not allow for the detailed consideration of the body (Jones, 2016). Our data supports this argument. While our data reveal disability is constructed in a social relational manner (Thomas, 2004), the data shows managing the leaky body – particularly heavy and unpredictable menstrual bleeding, affected women at work. The impairment effects of pain, blood and (perceived) odour need to be taken into account into the management and support of women and disabled people at work.

Theorising the disabled worker in these ways offers potential opportunities for a new workplace‐focussed dialog between medical sociology and disability studies. For instance, through the adoption of non‐Cartesian perspectives, as advocated by Price (2015) on the lived experiences of disabled and women workers, greater emphasis can be placed on the interdependencies between the expertise and creativity of their thinking, on the one hand, and the inclusiveness of their leaky, unruly and impaired bodies, on the other hand. This is important because workplaces like universities cannot have one without the other, so would be better off seeking to accommodate diverse bodies at work by prioritising skill retention and progression, instead of promoting competitive and impossibly idealistic standards. Where disabled and leaky workers are defined in opposition to ableist notions of workplace presence and performance, they are placed in a position where they must undertake near‐constant resistance and labour to mask and contain their ‘imperfections’. This labour penalty could be given greater recognition as something creating a starting point of disadvantage for disabled employees, which will be reinforced and exacerbated by other relationships, practices and policies merely assuming the employee will do well despite their impairment.

The Social Relational Model of disability used to frame this study builds on an extends the social model by allowing greater insights into impairment effects involving particular debilitating symptoms, and long‐term conditions involving experiences of pain, fatigue and shame. Disabled employees are oppressed by those without ‘impairments’, but the impacts of the impairment itself on a disabled individual are still also acknowledged (Thomas, 2004). Social relational approaches to disability, such as that in the current study, also highlight and tease out how workplace practices and ideals give rise to nuanced employment relationships, involving disabling barriers to ‘doing’ and ‘being’ an employee at work (Cologon, 2016; Jammaers et al., 2016; Sang et al., 2016). A critical disability studies approach could be applied to further research on this topic as it also incorporates the body and impairment and is interdisciplinary (Reaume, 2014), this would result in a novel disciplinary approach to employment focussed studies.

The current research takes the leaky body (Shildrick, 1997) as a feminist concept and embodied issue and develops it more in conjunction with disability studies by highlighting the (mis)management and (lack of) support available for those managing physical unruliness or leakiness of their bodies in the workplace. The findings contribute to a growing body of organisational literature recognising the body as a key material aspect of how labour is organised, and how it affects gendered lived experiences of work for specific workers; including women experiencing menopause (Beck et al., 2021; Jack et al., 2018), pregnancy (Gatrell, 2013), and menstruation (Sang et al., 2021). However, where existing research has often focussed more on the organisational responses or individual subjectivity of women, the current study develops a more specific focus on the management of material effects of bodily fluids.

Another implication of the current research concerns the value of conceptualising the under‐ and unemployment of disabled people with leaky bodies in the workplace in terms of a nexus of ‘dirty work’ and ‘body work’. In existing literature, dirty work and body work have often been treated separately in terms of different types of taint or stigma attached to work activities, and distinct forms of work activities involving attending to one’s own body and/or the bodies of others (Wolkowitz, 2002). Here, in studying the experiences of academics with troubling menstruation experiences and gynaecological health conditions, dirty and body work are central to the experiences of leakiness as employees are disabled by the workplace requirements made of them to manage, mask or contain their bodily fluids and minimise their pain and fatigue.

Although dirty work and body work are typically studied in relation to health and social care workplace settings (Twigg et al., 2011), these intersecting issues concerning feminism, disability studies, medical sociology and dirty body work are likely to be relevant to many other workplace and employment contexts, such as the academic ones analysed here. Workplace practices have the power to affect definitions of bodies, gendered divisions of labour, normative forms of social interactions, and the availability of facilities for supporting the disposal of bodily fluids. The body work of workers managing their own bodies demands greater attention to important temporal, spatial and gendered elements across different contexts, stages of life and environments.

The current findings suggest managing a leaky body can look different in the workplace than in the relative privacy and comfort of the home. Workplace pressures, norms, and practices can create disabling barriers such that bodily functions cannot be as conveniently and accessibly addressed as they might otherwise be domestically. Furthermore, considerable shame, stigma and taboo still surrounds women’s reproductive systems and menstrual blood (Gatrell, 2019) giving rise to additional social and relational barriers to authentically ‘being’ a worker with healthy self‐esteem and an identity that is not threatened or misunderstood (Cologon, 2016).

Importantly, greater sociological attention can continue to be given not just to work where other people’s bodies are managed, but to large diverse workforces where, at the intersection of medical impairment and social relational disability experiences, many people are engaged in the challenges of managing one’s own leaky body at work.

## AUTHOR CONTRIBUTIONS

**Jennifer Remnant**: Conceptualization (Equal); Data curation (Equal); Formal analysis (Equal); Investigation (Equal); Methodology (Equal); Writing – original draft (Lead); Writing – review & editing (Lead). **Katherine Sang**: Conceptualization (Lead); Data curation (Equal); Formal analysis (Equal); Funding acquisition (Lead); Investigation (Equal); Methodology (Equal); Project administration (Lead); Supervision (Lead); Writing – original draft (Supporting); Writing – review & editing (Equal). **Katriona Myhill**: Conceptualization (Equal); Data curation (Equal); Formal analysis (Equal); Investigation (Equal); Methodology (Equal); Project administration (Equal); Writing – original draft (Supporting); Writing – review & editing (Supporting). **Thomas Calvard**: Writing – review & editing (Supporting). **Sushila Chowdhry**: Conceptualization (Supporting); Writing – original draft (Supporting); Writing – review & editing (Supporting). **James Richards**: Writing – review & editing (Supporting).

## Data Availability

The data that support the findings of this study are available on request from the corresponding author. The data are not publicly available due to privacy and ethical restrictions.

## Study 1

The aim of Study 1 was to understand the experiences of disabled academics in relation to HRM practices. A qualitative approach was taken, involving semi‐structured interviews and electronic communications. Interviewees were recruited using convenience sampling; via social media and circulation of calls for participants by universities’ heads of schools and disability services in the UK. Rather than sample employees with a single impairment or syndrome of impairments (e.g., Richards, 2012), the focus of the research was to trace commonalities and differences across a maximally wide range of impairments affecting work and employment participation (Jammaers & Zanoni 2020). Each participant was provided with a consent form with study details and interview questions. To accommodate the complex needs of some participants, interviews took one of two forms: (1) twenty‐two synchronous telephone or Skype interviews; or (2) fifty‐three electronic interviews via email or online, shared documents for example, Google Docs. The latter data collection method meant participants could take part while allowing for impairment effects. Seventy‐five people participated in the research and constituted the final sample.

Fifteen synchronous interview participants were white women (68%), but the sample also included a range of ethnicities, disciplinary backgrounds, ages, career stages and impairments. Participants came from science, engineering, medicine, mathematics, social sciences and humanities, and occupied a diverse range of research and teaching roles, including laboratory work, fieldwork and desk‐based research. Participants held a variety of hourly‐paid, voluntary, independent, fixed‐term and open‐ended contracts. Impairments reported included: neurodiversity (autism spectrum disorders, dyslexia, dyspraxia), mental health conditions, mobility impairments, progressive neurological conditions, gynaecological conditions, traumatic brain injury, coordination disorders and muscular conditions. Participants had visible and hidden impairments, as well as acquired and/or lifelong health conditions. Refer to Table 2 for the synchronous participants’ characteristics in summary form.

Regarding asynchronous interviews/e‐responses, forty‐five were from women (85%), thirty‐four in social sciences (64%) and thirty‐four worked full‐time (64%). Impairments and health conditions included chronic health conditions (e.g., bowel conditions), neurological conditions (e.g., multiple sclerosis), musculoskeletal conditions, autoimmune diseases (e.g., arthritis) and mental health conditions. Most participants indicated one diagnosis, although fourteen (26%) indicated co‐morbidity (e.g., physical with mental health conditions). Twelve had left higher education due to impairment‐related issues (23%), although continued to work in related educational and research roles.

For synchronous interviews research ethics were reiterated at the start of the interview, and detailed field notes taken to aid analysis. The semi‐structured interview format allowed for flexibility, where the main substantive questions asked about:

(1) Career history
(2) ‘Impairment’ history
(3) Experiences of work and HRM in relation to their impairment or disability
(4) How organisations could better support them and their careers

Synchronous interviews lasted between 55 and 150 min (average 60 min). Those who completed the questions electronically were provided with the same questions. Recordings were professionally transcribed. For the electronic interviews, each participant returned their answers at a time convenient to them, and follow‐up questions were also asked via email.

The primary data were collected by the lead author, herself a disabled academic, noting research into disabled people’s experiences should be disability‐led (Barnes, 1996). The second author of the research is not a disabled academic but came from a perspective of research interest in the social construction of diversity and well‐being in relation to HRM. In addition, the second author has experienced chronic ill health. The third author is not a disabled academic and does not have a long‐term health condition.

### Analysis

Transcripts were read carefully to identify emerging themes; to understand how disability is socially and relationally constructed in interactions between academics and other staff, as well as through encounters with HRM practices and any discrepancies between ‘intended’ and ‘implemented’ practices (Piening et al., 2014).

Themes in the data were coded deductively and inductively in iterative cycles (Fereday & Muir‐Cochrane, 2006). Deductively, in terms of interview content’s relevance to the set question areas; HRM practices, disability studies, and studies of inequalities in work and employment. Inductively, in terms of common and unique uses of language, identity constructions, and experiences of contexts relevant to working in academia and universities. The lead author coded independently, meeting regularly with the other authors to discuss and refine coding frames and re‐code accordingly in several iterative rounds, until agreement and saturation was reached.

## Study 2

The aim of study two was to explore how academics manage menstruation and gynaecological health in the workplace. In order to explore the lived experience of individuals working in academia and whom have a gynaecological health condition, a qualitative approach using semi‐structured interviews was adopted. These testimonies assist in improving understanding of the relationship between women’s health and employment and the issues faced by women and non‐binary people in relation to the management of menstruation and gynaecological health in the workplace.

The study involved interviews with 23 women who currently, or had recently, worked in academia and who identified as having a gynaecological health condition. Given the exploratory nature of the study, a purposive sampling approach was deemed appropriate with participants recruited on the basis that they satisfied the criteria necessary for participation.

Interviewees were recruited via a follow up email following completion of a survey related to women academic’s experiences of managing menstruation and gynaecological health at work. Participants were asked upon completion of the initial survey to provide their contact details if they would be happy to take part in an interview and were selected for interview on the basis that they identified as having a gynaecological health condition and were currently or recently employed within an academic position in the UK. For summarised information of the participant characteristics please refer to Table 3.

Interviews were conducted via Skype or over the telephone and were recorded in order to aid with the accurate analysis of the interview data. Consent to record interviews and to share interviews with a professional transcription company was obtained prior to undertaking the interview and recordings were stored on secure, University servers.

The study received full ethical approval from the author’s institution and participants were also informed, prior to participation, of the full purpose of the study and their right to withdraw at any point. In addition, participants were assured of their anonymity and interview transcripts were anonymised to protect participant’s identities.

### Analysis

Transcripts were uploaded to Nvivo 12 to assist with the organisation and analysis of data. Data was coded and analysed using a thematic approach with a number of key themes identified including; the normalisation of pain; silencing/being silenced; the structure of the working day; disability; blood and; inadequate workplace policies.

## Study 3

The purpose of Study three was to explore the experiences of those involved in the support and management of disabled academics and academics with long term health conditions.

### Interview data

Purposive sampling was adopted to identify key stakeholders who could contextualise the development and implementation of disability‐related policy. Thirty‐five participants were recruited from three research‐intensive universities across Scotland selected due to their stated commitment to disability inclusion. Fourteen participants were in positions of line management, including members of university executives and heads of departments. Two of these participants had specific responsibilities relating to EDI. Five participants were Human Resources staff, one of whom had a specific EDI role. Two were members of Estates staff, one worked in university Health and Safety, and three worked in research administration or research‐only roles. Six participants were UCU [UK trade union for academic staff] branch members involved in case work at their employing universities. Four participants held disability specific roles supporting students and staff. For participant pseudonyms and information please refer to Table 4.

Of the participants, 35 participated in semi‐structured interviews remotely or in person with the lead author and one participant responded to questions via email. Interviewees were asked about their experiences of supporting disabled employees and what policies or legislation they drew on to provide that support. Interviews were transcribed and anonymised for analysis.

### Analysis

Interview transcriptions and policy data were uploaded to NVivo 12 for data management and to facilitate comparison between and across the data (Hutchison et al., 2010). Researcher familiarity with Equality, Diversity and Inclusion (EDI) and HRM practices allowed for thematic coding, alongside drawing on Bacchi’s work discussing the thematic analysis of policy documents (2009). The analytical protocol involved a combination of inductive and deductive thematic analysis, which is an approach utilised by authors exploring policy documents alongside qualitative data (see Fereday & Muir‐Cochrane, 2006).

The lead author read, and reread data generated during fieldwork and analysis to ensure that the developing themes were grounded in original data. The primary objective throughout data collection was to collect and then represent the subjective viewpoints of the interviewees who shared their experiences of managing or supporting disabled colleagues, and the organisational policy context that informed these experiences.

The study received full ethical approval from the lead author’s host institution at the time of data collection.
